# Population structure and genome-wide evolutionary signatures reveal putative climate-driven habitat change and local adaptation in the large yellow croaker

**DOI:** 10.1007/s42995-023-00165-2

**Published:** 2023-04-07

**Authors:** Baohua Chen, Yulin Bai, Jiaying Wang, Qiaozhen Ke, Zhixiong Zhou, Tao Zhou, Ying Pan, Renxie Wu, Xiongfei Wu, Weiqiang Zheng, Peng Xu

**Affiliations:** 1 Fujian Key Laboratory of Genetics and Breeding of Marine Organisms, College of Ocean and Earth Sciences, Xiamen University, Xiamen, 361102 China; 2 National Key Laboratory of Mariculture Breeding, Ningde Fufa Fisheries Company Limited, Ningde, 352000 China; 3 State Key Laboratory of Marine Environmental Science, Xiamen University, Xiamen, 361102 China; 4 Institute of Biotechnology, Fujian Academy of Agricultural Sciences, Fuzhou, 350000 China; 5 College of Fisheries, Guangdong Ocean University, Zhanjiang, 524088 China; 6 Ningbo Academy of Oceanology and Fishery, Ningbo, 315012 China

**Keywords:** Climate adaptation, Habitat change, Large yellow croaker, Phylogeographic structure

## Abstract

The large yellow croaker (*Larimichthys* *crocea*) is one of the most economically valuable marine fish in China and is a notable species in ecological studies owing to a serious collapse of wild germplasm in the past few decades. The stock division and species distribution, which have important implications for ecological protection, germplasm recovery, and fishery resource management, have been debated since the 1960s. However, it is still uncertain even how many stocks exist in this species. To address this, we evaluated the fine-scale genetic structure of large yellow croaker populations distributed along the eastern and southern Chinese coastline based on 7.64 million SNP markers. Compared with the widely accepted stock boundaries proposed in the 1960s, our results revealed that a climate-driven habitat change probably occurred between the Naozhou (Nanhai) Stock and the Ming-Yuedong (Mindong) Stock. The boundary between these two stocks might have shifted northwards from the Pearl River Estuary to the northern area of the Taiwan Strait, accompanied by highly asymmetric introgression. In addition, we found divergent landscapes of natural selection between the stocks inhabiting northern and southern areas. The northern population exhibited highly agminated signatures of strong natural selection in genes related to developmental processes, whereas moderate and interspersed selective signatures were detected in many immune-related genes in the southern populations. These findings establish the stock status and genome-wide evolutionary landscapes of large yellow croaker, providing a basis for conservation, fisheries management and further evolutionary biology studies.

**Supplementary Information:**

The online version contains supplementary material available at 10.1007/s42995-023-00165-2.

## Introduction

The large yellow croaker (*Larimichthys* *crocea*) is a marine fish that lives in the northwestern Pacific, generally in temperate nearshore seas and estuaries. The great abundance of fishery resources made it an economically important species in Northeast Asia, including China, Korea, and Japan. However, due to serious overfishing, wild populations of this species have suffered a serious collapse since the 1970s (Liu and De Mitcheson 2008; Orleans and Davidson 1980). Cultured fish rapidly took the place of ocean-caught fish in the market. The Chinese marine culture industry currently produces over 250,000 tons of large yellow croaker, ranking first among mariculture fish species (China Fishery Statistical Yearbook 2021). Despite rapid development in the past decades, the industry has faced considerable challenges related to germplasm recession, manifested as declines in growth rate, feed conversion ratio, and disease resistance, which may be caused by low genetic diversity in cultured populations (Wang et al. 2012).

Owing to the important implications for ecological protection, germplasm recovery, and fishery resource management, the population structure of large yellow croakers has been a highly controversial topic from the 1960s to the present. In the 1960s, some researchers proposed that there are three main large yellow croaker stocks along the Chinese coastline based on morphological data: Naozhou stock (NZ, distributed in the west South China Sea), Min-Yuedong stock (MYD, distributed in the eastern South China Sea and Taiwan Strait), and Daiqu stock (DQ, distributed in the East China Sea) (Tian et al. 1962; Xu et al. 1963). However, this proposition has been challenged. For example, there is evidence that large yellow croakers throughout the South China Sea belong to the same stock (Chen and Xu 2012; Li et al. 2013; Xu and Chen 2011). Despite the application of molecular genetic markers in recent years, the population structure of the large yellow croaker has not been clearly resolved. For example, an unweighted pair-group method with arithmetic mean (UPGMA) tree based on eight strictly selected simple sequence repeats (SSRs) supported the division of the species into NZ, MY, and DQ stocks (Lin et al. 2012). However, another study based on SSR markers did not identify genetic differentiation among geographical populations of large yellow croakers (Wang et al. 2012). Wang et al. (2012) used ten SSR markers that were initially developed for the small yellow croaker. Although these two species are closely related, levels of polymorphism of these marks in large yellow croaker populations were lower than those reported by Lin et al. (2012), which might explain the lack of phylogenetic resolution.

Single nucleotide polymorphisms (SNPs), as a new generation of genetic markers, are plentiful in the genome and easy to access. Population structure has been assessed based on genome-wide SNPs in many fishes, such as sharks (Junge et al. 2019), coral reef fishes (Picq et al. 2016), and sea bass (Zhao et al. 2018). In addition, high-density and genome-wide SNPs can be used to scan for evidence of fine-scale local adaptation, which may facilitate studies of the genetic basis for long-term microevolution in populations inhabiting different sea areas.

Herein, we employed next-generation sequencing to detect genome-wide SNP markers in large yellow croaker populations distributed along the eastern and southern Chinese coastline. We evaluated the fine-scale genetic structure and patterns of introgression and isolation in the species. In addition, we detected signatures of selection across the genome to investigate the mechanism underlying adaptive evolution in populations in different habitats.

## Results

### Sequencing and SNP discovery

**Fig. 1:**
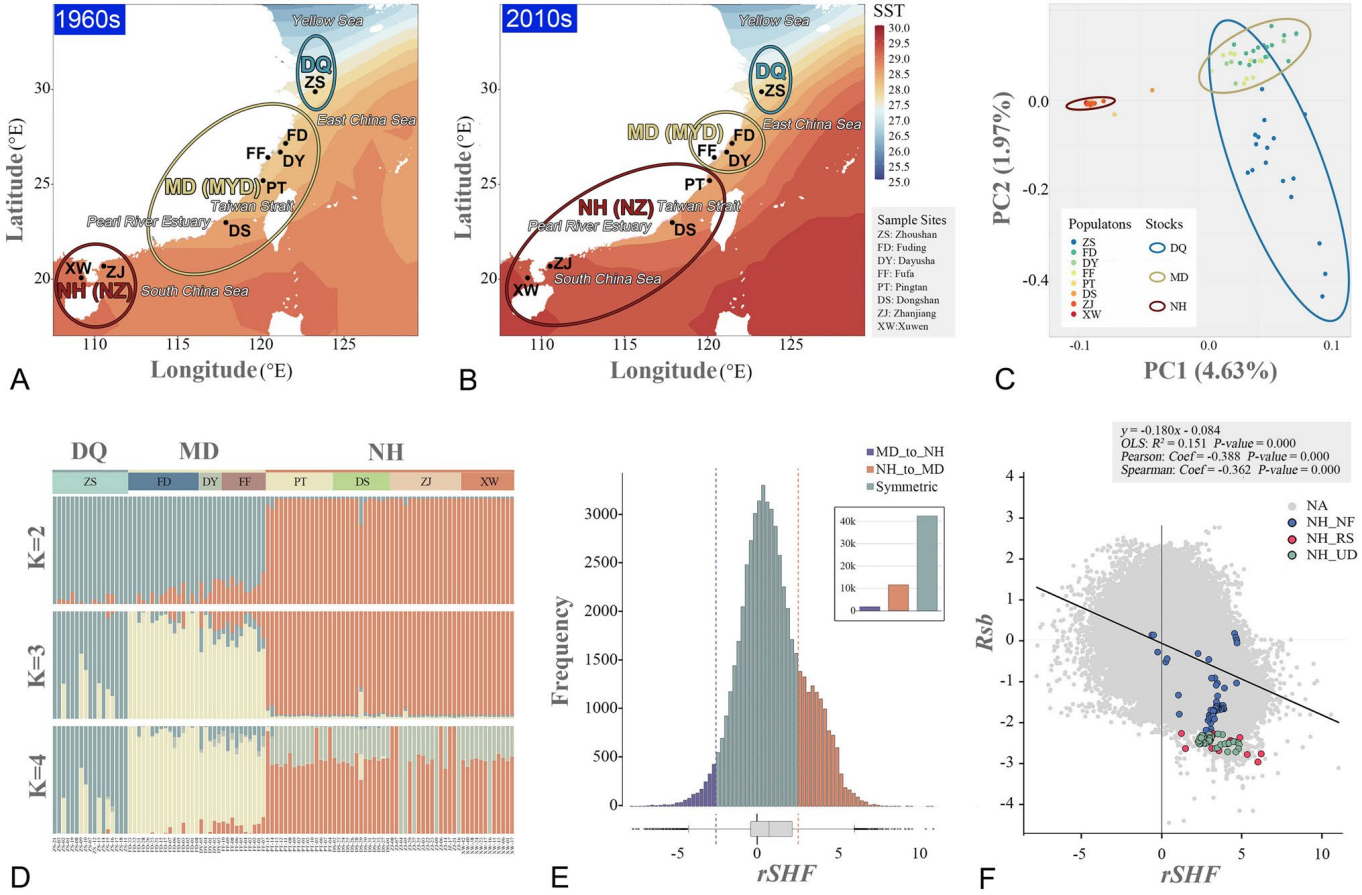
Population genetic analyses reveal a putative habitat change between large yellow croaker stocks. Geographical overview of sample sites and distribution of different stocks in the 1960s (**A**) and 2010s (**B**). Stock distributions are indicated by translucent ellipses covering sample sites. Colors indicate the annually averaged sea surface temperatures (SST). **C** Principal component analysis (PCA) based on SNP data reveals that large yellow croaker populations can be divided into three stocks. Confidence ellipses were drawn at 95% significance levels. **D** Ancestral admixture among geographical populations of large yellow croaker. The number of populations was set to 2–4. **E** The relative shared haplotype frequency (rSHF) index reveals asymmetric introgression between NH and MD stocks. Positive rSHFs indicate stronger introgression from the NH stock to the MD stock. The dashed lines in the positions of |rSHF| = ± 2.5 indicate the threshold used to detect asymmetric introgression. The upper-left bar plot shows the total number segments associated with symmetric and asymmetric introgression. **F** Scatter plot shows a significant negative linear correlation between rSHF and Rsb. Colors of dots represent the three types of natural selection in the NH stock: nearly fixed (NF), recently selected (RS), and undetermined (UD)

We collected 104 large yellow croakers from 8 sites (Fig. 1A; Table S1 in Supplementary file 1), including Zhoushan (ZS), Fuding (FD), Fufa (FF), Dayushan (DY), Pingtan (PT), Dongshan (DS), Zhanjiang (ZJ) and Xuwen (XW). We conducted genome resequencing and initial variant discovery based on these samples. We obtained 1191.42 Gbp of raw data (Table S2 in Supplementary file 1) and 8.25 million high-quality SNPs. Since elevated levels of linkage disequilibrium (LD) could result in the overrepresentation of some regions in the analysis of population structure, we used LD-based SNP pruning and generated a subset of SNPs containing 7.64 million markers (Fig. S1 and Table S3 in Supplementary files). All population genetic analyses were based on this pruned SNP set.

### Genetic diversity and population structure

We estimated basic population genetic parameters to determine the status of each population (Table S4 in Supplementary file 1). We first employed the *K*-means clustering method to partition values into two groups for each parameter. Then, we used one-way analysis of variance (ANOVA) to compare the two groups. For observed heterozygosity (*H*_O_) and the inbreeding coefficient (*F*), populations were partitioned into Zhoushan (ZS), Fuding (FD), and Zhanjiang (ZJ) vs. the other five populations. The ZS-FD-ZJ group had significantly lower *H*_O_ values and higher *F* values than the other group (*P* = 0.00445 and 0.00440, respectively), indicating higher levels of inbreeding. Based on expected heterozygosity (*H*_E_) and nucleotide diversity (*π*), populations were partitioned into FD and ZJ vs. the other six populations. The FD and ZJ populations had significantly lower *H*_E_ and *π* values than that of other populations (*P* = 0.00184 and 0.00384, respectively). These results suggest that large yellow croakers inhabiting these areas (i.e., ZS, FD and ZJ) have relatively low genetic diversity.

We then investigated the genetic structure of these populations via multiple methods. A principal component analysis (PCA) revealed that the sampled large yellow croakers can be divided into three distinctive stocks, i.e., Nanhai (NH), Mindong (MD) and Daiqu (DQ) (Fig. 1C). The NH stock was distributed in the South China Sea and the Taiwan Strait. The MD stock included three populations distributed near the Taiwan Strait: FF, DY, and FD. The DQ stock was formed by the ZS population. The genetic structure of these populations was clearly supported by ancestry proportions (Fig. 1D; Fig. S2 in Supplementary file 2). The *K*-value was set to 2 initially and updated by adding 1 for each repetition. When *K* = 2, the DQ stock and MD stock shared the same ancestry, while the NH stock was assigned to a separate group. In the model with *K* = 3, each of the three stocks identified by PCA had a unique hereditary constitution. And no additional effective information can be provided when set *K* greater than 3. Mixed ancestry was less common in the NH stock than in the other stocks. Gene introgression was generally in the direction of southern to northern populations. A phylogenetic tree constructed based on polymorphic SNPs using the maximum likelihood (ML) algorithm also revealed a very clear population structure coinciding with the results of the PCA and ancestry proportion analysis (Fig. S3 in Supplementary file 2).

### Genome-wide introgression pattern between the NH and DQ stocks

Shared haplotypes, also known as identity-by-descent segments, are valid indicators of introgression events among populations and stocks (Nagata et al. 2007). We identified 1.32 million shared haplotypes with a mean length of 19.659 kbp between all pairs of populations. The MD stock had much more inner-shared haplotypes than the NH stock, confirming their high level of inbreeding (Fig. S4 in Supplementary file 2). We identified a moderate-to-strong negative correlation (Spearman coefficient = − 0.706 and *P* = 2.691E−5) between the total length of shared haplotypes and position along the coastline. Populations closer to the two ends of the habitats (ZS and XW), in general, showed longer shared haplotypes (apart from ZJ and FD).

After dividing the genome into 25 kbp sliding windows (matching the windows used for the detection of selection), 55,694 windows overlapped with shared haplotypes between the PT and FF populations (the two adjacent sample sites flanking the boundary between the NH and MD stocks, Table S5 in Supplementary file 1). The time since the habitat changed is insufficient for the spread of introgressed segments to other sites in the new stock. Therefore, shared haplotypes in these segments should be more frequent within the original stock. Based on this concept, we determined the direction of introgression events by comparing the normalized shared haplotype frequencies (*nSHFs*) in the NH and MD stocks. The NH stock had an average *nSHF* that was 2.7 times higher than that of the MD stocks in the PT-FF shared haplotypes. Among these, 11,585 windows had log_2_-transformed relative SHFs (rSHFs) higher than 2.5, implying a higher chance that they originated from the NH stock. However, only 1763 windows had rSHFs lower than − 2.5 (Fig. 1E).

Moreover, we found that the rSHF values had an appreciable linear correlation with Rsb (*R*^2^ = 0.151) but not with Fst or Pi (Fig. 1F; Figs. S5, S6 in Supplementary file 2). In addition, both Pearson and Spearman coefficients indicated that there is a moderate negative correlation (Pearson coef. = − 0.388 and Spearman coef. = − 0.362) between Rsb and rSHF (Fig. 1F). Among 173 windows identified as NH-RS PSRs and associated with introgression between PT and FF populations, only nine had rSHF values less than 2.5, whereas no DQ PSR was associated with such introgressions.

### Isolation among large yellow croaker populations

Taking advantage of the high density of SNP markers, we calculated the genome-wide average fixation index (Fst), which indicates the degree of genetic differentiation between a pair of populations. Pairwise Fst values were 0.0356 ± 0.0212 (range, 0.0023–0.0626; Fig. S7 in Supplementary file 2).

**Fig. 2:**
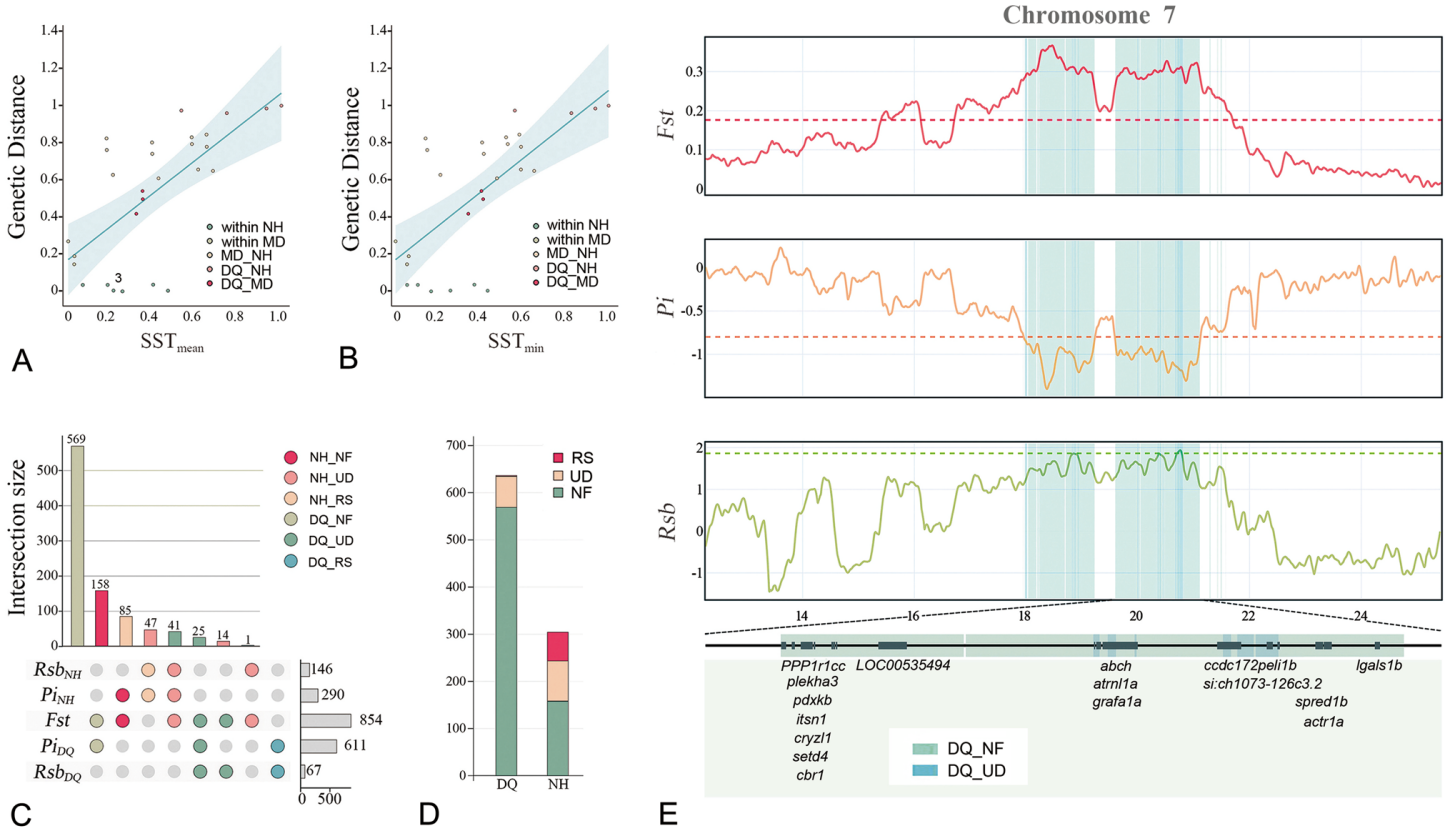
Isolation pattern and genome landscape of selective signatures of large yellow croaker stocks. **A** OLS regression shows a strong linear correlation between the genetic distance and the differences in annual mean SSTs (SST_mean_). Colors represent comparisons across or within different stocks. **B** OLS regression shows a strong linear correlation between genetic distance and annual minimum SST (SST_min_) differences. Colors represent comparisons across or within different stocks. **C** UpSet plot shows how PSRs were identified and classified. The combination matrix in the lower left shows all possible intersections of the five sets of outlier genomic regions. The horizontal bar plot on the lower right shows the sizes of these sets. The bars above the combination matrix indicate the numbers of outliers belonging to each intersection. **D** Bar graph shows the difference in the classification of PSRs between the DQ and NH stocks. **E** Signatures of selection around the longest PSR (highlighted by a pair of black dashed lines). Colored dashed lines indicate the thresholds for outlier identification. Magnified view at the bottom shows positively selected genes (PSGs) located in this PSR

Isolation-by-distance (IBD) and isolation-by-environment (IBE) are the two most common patterns explaining genetic distances in wild animal populations (Sexton et al. 2014), referring to the accrual of local genetic variation dispersed under geographical and environmental constraints, respectively. We modeled genetic differences based on the distance along the coastline (*D*_csl_), the “as the crow flies” distance (*D*_crf_), the distance along latitudes (*D*_lat_), and the difference by maximum, minimum, and mean SST (SST_max_, SST_min_, and SST_mean_) using ordinary least squares (OLS) to identify significant patterns among large yellow croaker populations (Table S7 in Supplementary file 1). The best IBD model was obtained using *D*_lat_ (*P* = 4.77E-04, *R*^2^ = 0.380) (Fig. 2A). The other two geographic factors, *D*_crf_ (*P* = 0.040, *R*^2^ = 0.152) and *D*_csl_ (*P* = 0.018, *R*^2^ = 0.197), did not show significant effects on the genetic differentiation of these populations (Table S8 in Supplementary file 1). Among climatic variables, both SST_mean_ (*P* = 8.80E−05, *R*^2^ = 0.452) and SST_min_ (*P* = 5.25E−05, *R*^2^ = 0.473) were important determinants of genetic differentiation among large yellow croaker populations (Fig. 2A, B; Table S8 in Supplementary file 1), whereas SST_max_ was not a significant predictor.

Although the magnitude of the effects of predictors differed, a simple comparison among significant predictors (*D*_lat_, SST_min_, and SST_mean_) might be unreliable due to the high multicollinearity (VIF = 60.31, 71.59, and 220.64, respectively). Hence, we employed three additional methods robust to multicollinearity to decompose the variance explained by the multiple linear regression (MLR) model. We employed LASSO (least absolute shrinkage and selection operator) regression and elastic net regression to rebuild the MLR model and disentangle the unique contributions of predictors in the MLR model by a weighting procedure (Nimon and Oswald 2013). The LASSO regression suggested that SST_min_ is the only meaningful predictor of genetic distance, with a coefficient of 0.765 and an *R*^2^ value of 0.461. A model with a slightly higher *R*^2^ value was obtained by Elastic Net regression combining SST_min_ and SST_mean_ (Table S9 in Supplementary file 1). These two predictors had positive coefficients (0.559 and 0.270, respectively) in the model. All weighted MLR indices also supported the importance of SST_min_ and SST_mean_. The validity, structure, and commonality coefficient ranked SST_min_ as the most effective predictor. SST_mean_ had the greatest importance among predictors by unique coefficients, general dominance, and relative weights (Table 1).

**Table 1:** Weighted multiple linear regression indices showing the dissection of the unique contributions of geographic and climatic predictors

Predictors	Validity coefficient	Structure coefficient	Squared structure coefficient	Unique coefficient
*D*_csl_	0.443	0.456	0.208	0.012
*D*_lat_	0.616	0.633	0.401	0
*D*_crf_	0.390	0.400	0.160	0.035
SST_max_	0.358	0.367	0.135	0.036
SST_min_	0.688	0.707	0.499	0.132
SST_mean_	0.673	0.691	0.478	0.136
Total	NA	NA	1.881	0.351

Predictors	Commonality coefficient	General dominance	Relative weight
*D*_csl_	0.185	0.124	0.124
*D*_lat_	0.380	0.173	0.184
*D*_crf_	0.116	0.126	0.111
SST_max_	0.091	0.097	0.107
SST_min_	0.341	0.203	0.205
SST_mean_	0.317	0.223	0.216
Total	1.430	0.946	0.947

### Genomic landscape of signals of positive selection

We identified 636 and 304 windows as positively selected regions (PSRs) in the DQ and NH stocks, respectively (Fig. 2C; Fig. S8, Table S10 in Supplementary files). We grouped windows by the type of selective pressure: nearly fixed (NF), recently selected (RS), and undetermined (UD). The number of NF PSRs was nearly 8.5 times that of RS PSRs (727 vs. 86). The merged NF PSRs were only approximately five times longer than RS PSRs (5.16 Mb vs. 9.95 Mb). The Kolmogorov–Smirnov test also suggested that RS PSRs showed a more uniform distribution than that of NF PSRs (*P* = 6.00E−25 vs. *P* = 8.72E−189). Notably, 89.47% of DQ PSRs were near fixation, whereas only 0.16% of PSRs were under recent positive selection. In contrast, 51.97% of NH PSRs were near fixation and 27.96% of NH PSRs showed evidence of recent positive selection (Fig. 2D). We further examined whether the five types of PSRs showed significant differences in other indicators of selection (excluding the DQ-RS PSR owing to the extremely small sample size). NF PSRs had significantly higher values than those of RS PSRs for all indices except Rsb, which is very sensitive to the onset time of selection (Fig. S9, Table S11 in Supplementary files).

### Candidate genomic regions and functional genes under selection

We found that the degree of overlap of RS PSRs was 63% higher than that of NF PSRs (0.463 vs. 0.284). Moreover, NF PSRs overlapped with 153 functional genes with a total overlap length of 2.29 Mb and a generic sequence proportion of 44.34%, whereas only 13 genes overlapped with RS PSRs, resulting in a generic sequence proportion of 27.80%. The largest PSR was a DQ-NF type PSR located between 17.97 and 19.24 Mb on Chromosome 7 with a length of 1.27 Mb (Fig. 2E). This PSR also had the highest gene density (33.07 gene/Mb) among all PSRs longer than 100 kb. These results suggest that stronger NF selective signatures are more unevenly and non-randomly distributed in the large yellow croaker genome and tended to be gathered around generic regions that directly affect fitness. Signatures of relatively weak RS selection were dispersed around the nongenic regions, providing slight advantages.

We identified 162 positively selected genes (PSGs) and divided them into six categories based on the type of PSR with which they overlapped (Fig. S10, Table S12 in Supplementary files). These included 107 and 55 unique DQ and NH PSGs. We then performed an overrepresentation analysis (ORA) followed by enrichment analyses to identify biological processes and clusters associated with climatic adaptation (Table S13 in Supplementary file 1). DQ PSGs were enriched in 16 functional categories, including 12 GO Biological Processes, 2 Reactome Gene Sets, and 2 KEGG pathways, involving 38 unique functional genes. It is worth noting that 10 of 16 terms were closely linked to the developmental processes, such as "regulation of angiogenesis" (GO: 0,045,765), "ear development" (GO: 0,043,583), and "pronephros development" (GO: 0,048,793). The development-related terms involved 25 DQ PSGs. Nearly half of these (12) were located on the longest PSR on Chromosome 7. The NH PSGs were enriched in nine GO Biological Processes and one KEGG pathway, involving 29 unique PSGs. Among these, five terms were related to immune system processes or responses to stimuli, such as “leukocyte differentiation” (GO: 0002521), “lymphocyte differentiation” (GO: 0030098), and "double-strand break repair" (GO: 0006302).

## Discussion

### Classification of wild large yellow croaker populations and recent habitat changes

Although the classification of wild large yellow croaker populations in coastal waters of China has been debated for a long time, this issue remains unresolved. By a detailed literature search, we identified two general views. The traditional division is the DQ-MYD-NZ three-stock system initially proposed by Tian and Xu in the 1960s (Tian et al. 1962; Xu et al. 1962, 1963) and confirmed by subsequent studies (Lin et al. 2012; Zhang et al. 2011). In contrast, many other researchers believe that only two stocks exist by combining the DQ and MYD stocks (Chen and Xu 2012; Chen et al. 2016; Li et al. 2013; Wang et al. 2012; Xu and Chen 2011). Our genome-scale population genetic analysis revealed at least three wild large yellow croaker stocks. Fish inhabiting the East China Sea and the Yellow Sea were divided into two distinct stocks based on genetic differentiation (Fig. 1C, D; Figs. S2, S3 in Supplementary file 2).

Most previous studies suggesting that large yellow croakers inhabiting the East China Sea and the Yellow Sea belong to the same stock are based on a very limited number of genetic markers, which may not be sufficiently powerful to evaluate genetic differentiation. Our results were not in complete agreement with the traditional division. It was initially proposed that two stocks inhabit the South China Sea and waters near the Taiwan Strait with the Pearl River Estuary as a boundary (Tian et al. 1962; Xu et al. 1962, 1963), whereas our results suggest that the boundary between these two stocks is the Taiwan Strait. Hence, we rename these the Nanhai (NH) and Mindong (MD) stocks. This disagreement in the boundary between stocks may be explained by the variation in morphological traits used by Tian and Xu to divide the stocks. Alternatively, it may be explained by a habitat shift over time. In the past few decades, the MYD wild germplasm resources collapsed after overexploitation in the coastal region of southeastern China, making the area between the Pearl River Estuary and Taiwan Strait an ecological space for large yellow croakers. In addition, global climate change resulted in a dramatic increase in sea surface temperature (SST) in China's coastal waters. In waters near the Taiwan Strait from the 1960s to 2010s, the mean monthly average SST increased by 0.5–0.9 °C (Fig. 1A, B). The large yellow croaker has a relatively limited tolerance to cold and hot environments, making it highly sensitive to climate change (Chen and Wu 2011; Gao et al. 2010; Liu et al. 2019; Qian and Xue 2016; Zhang et al. 2002). Therefore, we suppose that climate change promoted the expansion of the NH (NZ) stock habitat to the space left by the MD (MYD) stock. It is widely accepted that the population distribution and habitat range of fish species are very sensitive to climate change (Jeppesen et al. 2010; Munday et al. 2012; Pinsky et al. 2019).

Local extinction events are common in fish. For example, the Aral Sea stock of ship sturgeon (*Acipenser* *nudiventris*) and the Adriatic Sea stock of beluga sturgeon (*Huso* *huso*) have become extinct (Birstein 1993; Birstein et al. 1997). The observed distribution of the MD stock is already very narrow. If the SST of adjacent waters north of the Taiwan Strait continues to increase at the present rate, the MD stock will either migrate to more northern areas or completely lose their habitat. Considering that the MD stock accounts for the vast majority of aquaculture production of large yellow croakers (China Fishery Statistical Yearbook 2021), the aquaculture industry will suffer considerable damage without introducing germplasm from the NH stock or effective breeding for heat tolerance.

### Asymmetric introgression between the NH and MD stocks provides evidence for recent habitat change

Our results revealed that genetic introgression across the boundary between the NH and MD stocks is highly directional (Fig. 1E, F). Over 6.5 times more segments introgressed from the NH to the MD stock than the reverse. Asymmetric introgression is frequently reported and is considered a result of habitat changes in many species, including mammalians (Levanen et al. 2018), birds (Peters et al. 2017), amphibians (Sequeira et al. 2020), and fishes (Sefc et al. 2019). This finding supports our hypothesis that the NH stock expanded their habitat and invaded the territory of MD stock in the past few decades.

Asymmetric introgression is more likely to occur when the genomic segments confer a selective advantage (Jezkova et al. 2013; Melo-Ferreira et al. 2005). We identified a low-to-moderate correlation between the rSHF and Rsb statistics (Fig. 1F). This finding demonstrated that the introgressed regions contain much stronger extended haplotype homozygosity signals in the NH stock than in the DQ stock. Possibly due to genetic hitch-hiking and the short time since introgression, the RS regions had a significantly higher level of asymmetric introgression than that in the NF and UD regions (Fig. 1F). A similar association was also discovered in European pigs for an extremely long introgressed region and improved reproductive traits (Bosse et al. 2014). A previous genome-wide association study identified five SNPs significantly associated with acute heat tolerance in the MD stock (Wu et al. 2021). Among these, three were close (< 25 kbp) to windows with positive rSHF values, indicating that the asymmetric introgression from the NH stock may confer better heat tolerance in the MD stock (Table S6 in Supplementary file 1). This also presents a challenge for the maintenance of the unique MD stock germplasm, particularly if introgression from the NH stock is further enhanced by global ocean warming.

### Isolation-by-environment instead of isolation-by-distance impacted genetic differentiation

The relative roles and importance of drift and selection, two common drivers of genetic differentiation, is a longstanding topic (Abdel-Haleem 2007; Nei 1987). This issue can be addressed by evaluating the correlation of the genetic distance with geographic distance and environmental dissimilarity (Jiang et al. 2019). Significant correlations with geographic distance and environmental dissimilarity suggest that genetic drift and natural selection, respectively, are important determinants of population structure (Barnes et al. 2016; Kawecki and Ebert 2004). However, it is not clear whether gene flow in the large yellow croaker follows a pattern of IBD or IBE. In this study, we modeled relationships between genetic distances and geographic distances in an IBD analysis, and with sea surface temperatures (SSTs) in an IBE analysis. Although a number of environmental factors, such as salinity, dissolved oxygen, and pCO_2_, impact fish physiology, continuous long-term and broad-scale data are often lacking for these factors. Moreover, temperature is widely considered the most vital environmental factor for fish population dynamics and distributions (Campana et al. 2020; Geraldi et al. 2019; Loeng 1989).

Although IBD has been identified as a very important pattern in many marine fishes, such as tupong (O'dwyer et al. 2021), grayling (O'dwyer et al. 2021), and red drum (Hollenbeck et al. 2019), the role of environmental heterogeneity in genetic differentiation was much greater than that of geographic distance in the large yellow croaker (Fig. 2A, B; Table S8 in Supplementary file 1). The only significant geographic factor, *D*_lat_, was falsely positive due to its extremely strong correlation to SST based on further MLR analyses (Table 1; Table S9 in Supplementary file 1). The large yellow croaker produces pelagic eggs (Li et al. 2021), so individuals drift for a long distance in the early life stages (Wang et al. 2021). The excellent swimming ability of mature fish allows them to migrate across physical barriers for feeding, spawning, and overwintering (Xu and Chen 2011; Zhang 2015). Since easy dispersal weakens the effect of IBD in some species (Crispo and Hendry 2005; Phillipsen et al. 2015), we suggest that this is also the primary reason for the substantial genetic differentiation among large yellow croaker populations without an obvious IBD pattern.

These results also suggest that genetic differentiation in large yellow croakers was highly related to the annual mean and minimum SST. To some extent, this finding is consistent with our previous hypothesis that temperature has driven the habitat alterations in different large yellow croaker stocks in the past few decades. Similar results supporting the greater role of IBE than IBD were obtained in other species, such as kelp bass (*Paralabrax* *clathratus*), Kellet's whelk (*Kelletia* *kelletii*), California spiny lobster (*Panulirus* *interruptus*), and Pacific ridley (*Lepidochelys* *olivacea*) (Rodriguez-Zarate et al. 2018; Selkoe et al. 2010). Moreover, Sexton et al. (2014) summarized 70 studies of various species and determined that IBE is more closely related to genetic difference than is IBD in the natural environment. Our findings also revealed that lethal cold in the winter had a much greater impact on patterns of population differentiation than that of hot weather in the summer. The DQ stock are located near the northmost end of the distribution of large yellow croaker, where the SST drops to 10 °C in the winter (Fig. 1A, B). Such a cold environment exerts intense stress in large yellow croakers (Qian and Xue 2016) and may drive the genetic divergence between the DQ stock and other populations.

### Divergent landscapes of natural selection promote adaptation to different environmental conditions

Our genome scan of signatures of selection revealed that natural selection drove the evolution of the DQ and NH stocks in two distinct ways. In the high-latitude DQ stock, intense selection pressure was largely confined to limited gene-enriched genomic regions, directly impacting fitness. Alternatively, natural selection tended to act more moderately and on more broadly distributed nongenic regions in the low-latitude NH stock. In addition, DQ PSRs had more SNPs that were close to fixation, whereas NH PSRs had more SNPs at intermediate frequencies due to positive selection (Fig. 2C, D; Figs. S9, S10 in Supplementary file 2).

Cold temperatures are naturally a more lethal challenge than hot temperatures for fish species (Beitinger et al. 2000) for various reasons, e.g., cold-induced lethargy, a gentler vertical temperature gradient in the winter (Lafond 1954), and more rapid changes of temperature introduced by cold currents (Zhu et al. 2015). Therefore, we can suppose that mortality in the winter exerts a strong selection pressure and effectively eliminates the near-neutral mutations in some specific genomic regions in high-latitude populations.

A review of insect studies revealed that cold resistance tends to evolve more readily than heat resistance (Addo-Bediako et al. 2000). A very similar phenomenon has also been found in marine species, including fish (Stuart-Smith et al. 2017). In regions related to cold resistance, rapid changes in allele frequencies and high LD occur during rapid population size expansions after lethal cold selection. However, complex genetic mechanisms of heat resistance and relatively weak heat-related selection results in genetic variation that will be mutually neutralized during admixture.

### Different biological processes and pathways involved in adaptive evolution

Apart from distinct patterns of selection, different biological processes and pathways were involved in differentiation between the DQ and NH stocks (Table S13 in Supplementary file 1). Winter mortality is size-selective and can exert strong selective pressure on animals, especially ectotherms, such as fish, in temperate regions (Brodersen et al. 2011; Callahan et al. 2021; Hurst 2007; Takegaki and Takeshita 2020). The DQ stock inhabits the coldest frontier of the distribution of the large yellow croaker, which almost certainly constrains its development and growth. Many studies have revealed that ectothermic species in cold environments evolve toward more rapid early development to exploit the rare periods suitable for development (Carbonell et al. 2021; Perkins 2012; Quinn et al. 2013; Shine et al. 2011). The enrichment of DQ PSGs highlights the wide range of development-related biological processes that may have been important for escaping from size-selective winter mortality. Similar results have been obtained in studies of other fishes (Jensen et al. 2008; Kavanagh et al. 2010; Laugen et al. 2003).

Enrichment analysis of NH PSGs showed that immune system processes played the most important role in local adaptation to tropical environments. This finding may reflect high levels of immune stress due to increased pathogen virulence (Mitchell et al. 2005), diversity (Karvonen et al. 2013; Luque and Poulin 2008), and abundances (Karvonen et al. 2013) in warm environments. The main pathogens in large yellow croakers include *Cryptocaryon* *irritans*, *Vibrio* *alginolyticus*, Large yellow croaker iridovirus, and *Ichthyodinium* *chabelardi* (Gleason et al. 2019), which have high incidences at high temperatures (Abdullah et al. 2017; Yang et al. 2021). Adaptative microevolution in immune system-related genes might improve fitness in the NH stock in tropical conditions.

## Conclusion

This study generated millions of genome-wide SNP loci to evaluate the genetic structure and evolutionary history of large yellow croaker stocks. Our results generally supported one of two widely accepted stock divisions, the DQ-MYD-NZ (DQ-MD-NH) three-stock system. However, the genetic structure and asymmetric introgression indicated that the boundary between the NH and MD stocks may have moved from the estuary of the Pearl River to the Taiwan Strait. Based on our IBE analysis, we deduced that this habitat change was closely associated with climate change in the past few decades. The substantially different landscapes of selective signatures from the northernmost and southernmost stocks presumably arose from differences in responses to long-term cold and heat stress.

## Materials and methods

### Sampling and sequencing

We collected 104 large yellow croakers from eight sites in eastern and southern Chinese coastal waters (Fig. 1A; Table S1 in Supplementary file 1). White muscles or fin rays were collected from fish anesthetized by 100 mg/L tricaine methanesulfonate (MS222) solution. Samples were lysed in SDS digestion buffer with proteinase K. DNA was extracted using a standard phenol–chloroform protocol. Whole-genome shotgun libraries with 350 bp insert sizes were constructed according to the manufacturer's instructions (Illumina, San Diego, CA, USA) and paired-end sequencing was performed using the Illumina NovaSeq 6000 platform with a read length of 2 × 150 bp. Read filtering was conducted using SolexaQA++ (version 3.1) (Cox et al. 2010). Reads with adaptor sequences or proportions of unknown/low-quality bases greater than 10% were removed. By high-throughput sequencing, 7.97 billion pairs of raw reads were generated with a total length of 1.19 trillion bases and an average read depth of 19.49 × per sample. After quality control, 1.17 T bases (98.36%) were retained for downstream analyses (Table S2 in Supplementary file 1).

### Variant discovery, genotyping, and filtering

Clean reads were aligned using BWA-MEM (version 0.7.17-r1188) (Li and Durbin 2009) with default parameters to the reference genome of large yellow croaker (Chen et al. 2019). Then, GATK (version 4.1.9) was used for base quality score recalibration, indel realignment, and duplicate removal. SNP discovery and genotyping were performed across all 104 samples simultaneously according to GATK Best Practices recommendations (Van Der Auwera and O'connor 2020). Since there were no verified SNP loci or genotyping data for the large yellow croaker genome, we applied hard filtering on the variant callset (De Summa et al. 2017). SNPs with low quality were marked using VariantFiltration with a compound filtering expression “QD < 2.0 || QUAL < 30.0 || SOR > 3.0 || FS > 60.0 || MQ < 40.0 || MQRankSum < − 12.5 || ReadPosRankSum < − 8.0” and were then removed using SelectVariants. An additional filtering step was performed using VCFtools (version 0.1.15) (Danecek et al. 2011) to remove multiallelic SNPs, with minor allele counts less than 2, missing genotype counts greater than 2, or minor allele frequency less than 0.05. The final SNP set was annotated using SnpEff (version 4.3t) (Cingolani et al. 2012).

### Population structure analysis

We firstly reduced the redundancy of the final SNP set using plink (version 1.90b6.16) (Purcell et al. 2007) with the parameter “–indep-pairwise 10 2 0.8”. We calculated basic statistics, including observed heterozygosity (*H*_O_), expected heterozygosity (*H*_E_), and *F* values, for each population using the R package hierfstat (version 0.5–7) (Goudet 2005). Nucleotide diversity (*π*) was calculated using VCFtools (version 0.1.15) (Danecek et al. 2011). The *K*-means method was used to partition the values into two groups for each statistic. Then, one-way ANOVA was used to assess the significance of differences between the two groups. Statistical analyses were conducted using the “SciPy” and “sklearn” Python modules. A PCA was performed using the R package "SNPrelate" (version 1.16.0) (Zheng et al. 2012) after transformation of the data from variant call format (VCF) to CoreArray Genomic Data Structure (GDS) format using the R package "gdsfmt" (version 1.22.0). Additionally, frappe (version 1.1) (Tang et al. 2005) was used to estimate the genetic ancestry of each sample, which is highly efficient when using high-density SNP genotype data. The maximum iterations of expectation maximization (EM) were set to 100,000 and the number of populations (*K*) was set from two to ten for each calculation. A ML phylogenetic tree was constructed based on all samples. Firstly, RAxML (version 8.2.12) (Stamatakis 2014) was used to build the initial tree with a nucleotide substitution model GTRCAT. Then RAxML-Light (version 1.1.1) (Stamatakis et al. 2012) was used for the final ML tree construction. A ML search convergence criterion was used in this step. Finally, the tree file in Newick format was fed into the iTOL (version 6.5.4) (Letunic and Bork 2019) webtool for visualization.

### Detection of shared haplotypes and direction of introgression

All SNP loci were phased using beagle (version 5.2) with default parameters, except for a window length of 4.0 cM and a window overlap of 0.5 cM. Then, pairwise shared haplotypes were extracted from each chromosome using RefinedIBD (version 17Jan20.120) with a window size of 10 cM, minimum reported LOD score of 3.0, minimum reported haplotype length of 0.025 cM, and trimmed length when calculating LOD score of 0.0025 cM. All shared haplotypes between individuals sampled from the same site were filtered.

The direction of introgression between the NH and MD stocks is determined based on a basic concept; given the short time since the habitat change, introgressed segments cannot yet spread to other sites in the new stock. Therefore, shared haplotypes on these segments should be more frequent within the original stock. The frequency of shared haplotypes (SHF) was estimated as described previously (Bosse et al. 2014), with minor modifications.

We divided the genome into 25 kbp windows with a step size of 5 kbp. If a window overlapped with a shared haplotype longer than 10 kbp, it was regarded an introgression event. The number of introgressions between all pairs of populations (sites) was calculated per window. As the total number of pairwise comparisons differed between the groups, these numbers were normalized, ranging from zero (no shared haplotype tract detected) to one (haplotype shared by all individuals within the group). Then, the direction of gene introgression across the boundary between the NH and MD stocks was determined by the relative shared haplotype frequency (rSHF) of introgression, which was calculated using the following equation:

$$\mathrm{rSHF}={\mathrm{log}}_{2}\frac{{\mathrm{cSHF}}_{\mathrm{NH}}/ {\mathrm{tSHF}}_{\mathrm{NH}}}{{\mathrm{cSHF}}_{\mathrm{MD}}/ {\mathrm{tSHF}}_{\mathrm{MD}}},$$

where cSHF_NH_ and cSHF_MD_ are the counts of all shared haplotypes within the NH and MD stocks, respectively. tSHF_NH_ and tSHF_MD_ are the total pairwise comparisons within the NH stock and MD stock, respectively. The ratio between cSHF and tSHF was also called the normalized shared haplotype frequency (nSHF).

Differences between nSHF_NH_ and nSHF_MD_ were evaluated by a parametric test (ANOVA) and two non-parametric tests (Kruskal–Wallis and Alexander–Govern approximation tests). All tests were carried out using the “stats” module of the "scipy" Python package (version 1.7.1) (Virtanen et al. 2020). The same module was used to calculate the Pearson and Spearman correlation coefficients for relationships between rSHF and natural selection parameters. The "ols" module in the "statsmodels" Python package (version 0.12.1) (Seabold and Perktold 2010) was used to build the ordinary least squares linear models with rSHF and natural selection parameters.

### Isolation-by-distance and environment analysis

The genetic distance Fst/(1 − Fst) was calculated using VCFtools (Danecek et al. 2011) (version 0.1.15). The “as the crow flies” distance between each sample pair was calculated directly from the longitude and latitude coordinates. To calculate the D_csl_, pre-processed OpenStreetMap data were obtained from Planet OSM (https://osmdata.openstreetmap.de) and transformed into "Mercator" projections using the R package "sf" (version 0.9.7). D_csl_ was modeled as the total length of diagonal lines of all 0.1 km grids passed through the coastline between two sites. SST values were obtained from the Kaplan Extended SST V2 dataset available at https://psl.noaa.gov/ (provided by the NOAA/OAR/ESRL PSL, Boulder, Colorado, USA). An in-house Python script was used to parse the NetCDF4 file under the "netCDF4" Python library (version 1.5.5.1). The annual SST_max_, SST_min_, and SST_mean_ were calculated after removing the first five outliers.

Ordinary least regression models between genetic distances and exogenous variables were built using Python libraries "statsmodels" (version 0.12.1). Regularized least squares (RLS) models were built using the "linear_model" module imported from the Python library "sklearn" (version 0.24.0) to deal with multicollinearity among variables when decomposing their contribution to genetic differentiation. RLS is a family of relatively new linear regression techniques with high tolerance to multicollinearity among variables. It has been widely used for variable selection by setting coefficients of less important variables to zero in machine learning, data mining, and bioinformatic approaches (Tibshirani 2011). It was recently introduced to solve problems of confounding and causality plaguing genome-environment association studies (Frichot et al. 2015). In addition, we also employed the R package "yhat" (version 2.0-3) (Nimon and Oswald 2013) to perform a weighting procedure to disentangle the unique contributions of predictors. The "booteval.yhat" function in this package was used to generate confidence intervals of all weighting MLR indices with 5000 bootstrap replicates. All R functions and scripts were executed under a Python interface embedded in R using the "rpy2" Python library (version 3.4.2).

### Scanning for genome-wide signatures of selection

Signatures of selection in the large yellow croaker genome were detected by comparing the DQ and NH stocks. The genome was divided into 25 kbp windows with a sliding length of 5 kbp. Various indices of positive selection were calculated within each window, including the fixation index (Fst), nucleotide diversity (*π*), extent of haplotype homozygosity (Rsb), Tajima's *D*, and composite selection score (CSS). The fixation index (Fst) and raw nucleotide diversity (*π*) were calculated using VCFtools (version 0.1.15) (Danecek et al. 2011). The logarithm of ratio between π values for the two populations (denoted Pi) between the DQ and NH stocks was subsequently calculated within each window as an index of positive selection. The extent of haplotype homozygosity (Rsb) was calculated for each SNP using the R package "rehh" (version 3.0.1) (Gautier et al. 2017) and then averaged in each window. Tajima's *D* index was calculated using plink (version 1.90b6.16) (Purcell et al. 2007). The CSS index was calculated based on Fst, Pi, and Rsb following a previously described method (Avalos et al. 2017). Fst, Pi, and Rsb were used to identify positively selected regions (PSRs) after applying the 95th percentile of each index as thresholds. The Rsb values are inflated when an allele extends the length of homozygosity in either population in the comparison. In this way, the Rsb index is very sensitive to recent positive selection. The Pi index, which indicates the difference in nucleotide diversity between two stocks, has similar sensitivities to selection. Therefore, we defined the overlap of outliers based on Pi and one of the other two indices as near-fixation (NF) and recently selected (RS) regions. However, we could not classify the PSRs identified by Fst and Rsb indices; accordingly, these were classified as undetermined (UD) PSRs. In addition, the CSS and Tajima's *D*, as unbiased indices, were used to compare the strength of natural selection among different types of PSRs. The mean and standard deviation of each index were compared among various types of PSRs by ANOVA followed by least significant difference (LSD) tests at a significance level of *P* < 0.05.

Identification and classification of PSGs were performed based on the type of PSRs overlapping genes. Gene Ontology, KEGG pathway, and Reactome Gene Set enrichment analyses were performed for each type of PSG using Metascape webtool (version 3.5) (Zhou et al. 2019).

## Supplementary Information

Below is the link to the electronic supplementary material.

Supplementary file 1. An Excel spreadsheet includes 11 supplemental tables (XLS 6460 KB)

Supplementary file 2. A Word document includes 10 supplemental figures (DOCX 2366 KB)

## Data Availability

The raw sequence data reported in this paper have been deposited in the Genome Sequence Archive (GSA) (Wang et al. 2017) in the National Genomics Data Center (NGDC) (National Genomics Data Center and Partners 2020), China National Center for Bioinformation/Beijing Institute of Genomics, Chinese Academy of Sciences (GSA Accession ID: CRA005688) that are publicly accessible at https://ngdc.cncb.ac.cn/gsa/browse/CRA005688.
