# Supplementary material for: Population structure and genome-wide evolutionary signatures reveal putative climate-driven habitat change and local adaptation in the large yellow croaker
Source: Mar Life Sci Technol. 2023 Apr 7;5(2):141–54. doi: 10.1007/s42995-023-00165-2 (PMC10232709; doi:10.1007/s42995-023-00165-2)

**Figure S1.** An ideogram shows the SNP density across the large yellow croaker genome.

**
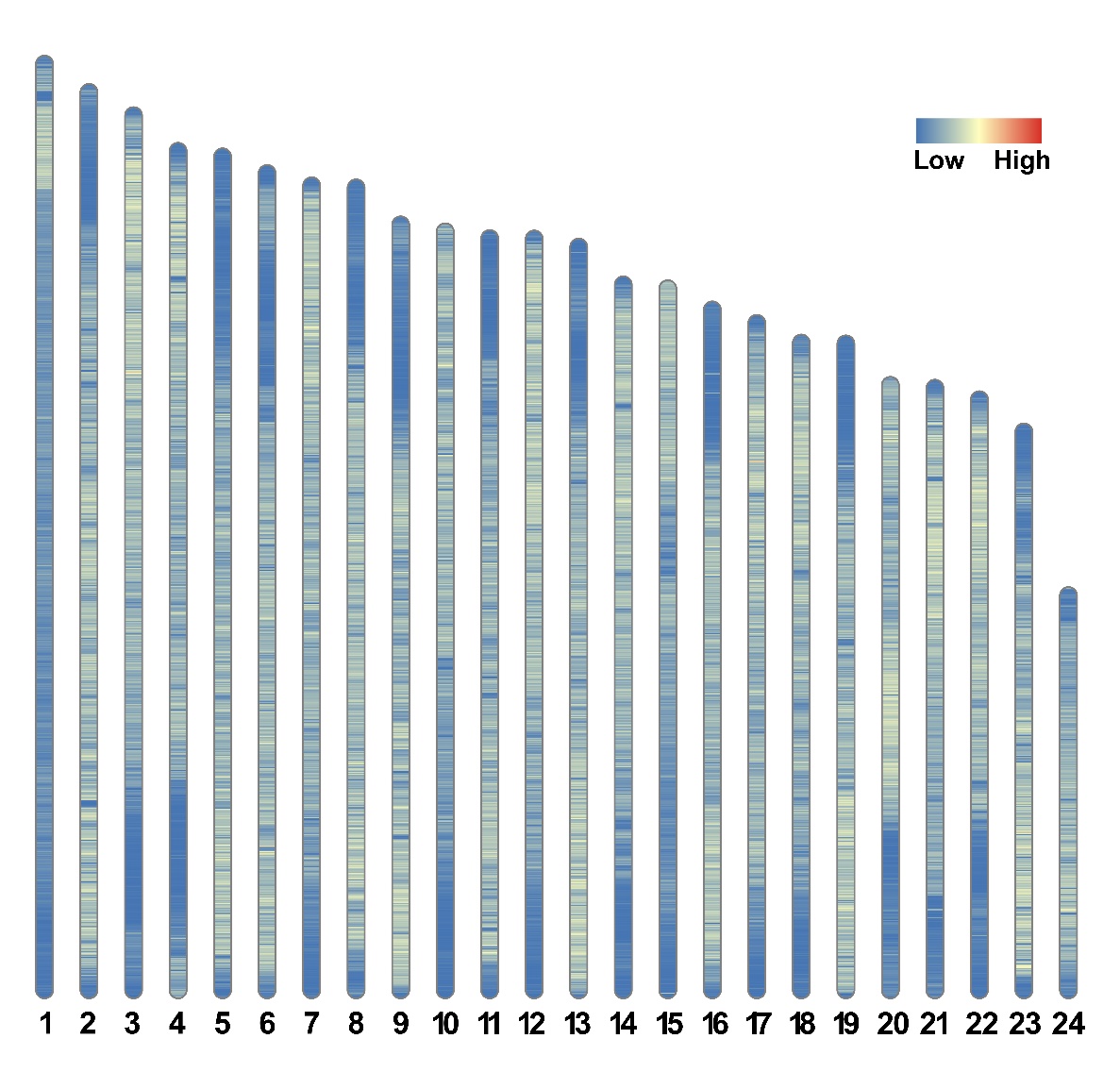
**

**Figure S2.** Ancestral admixture among geographical populations of large yellow croaker estimated when K was set to ten.


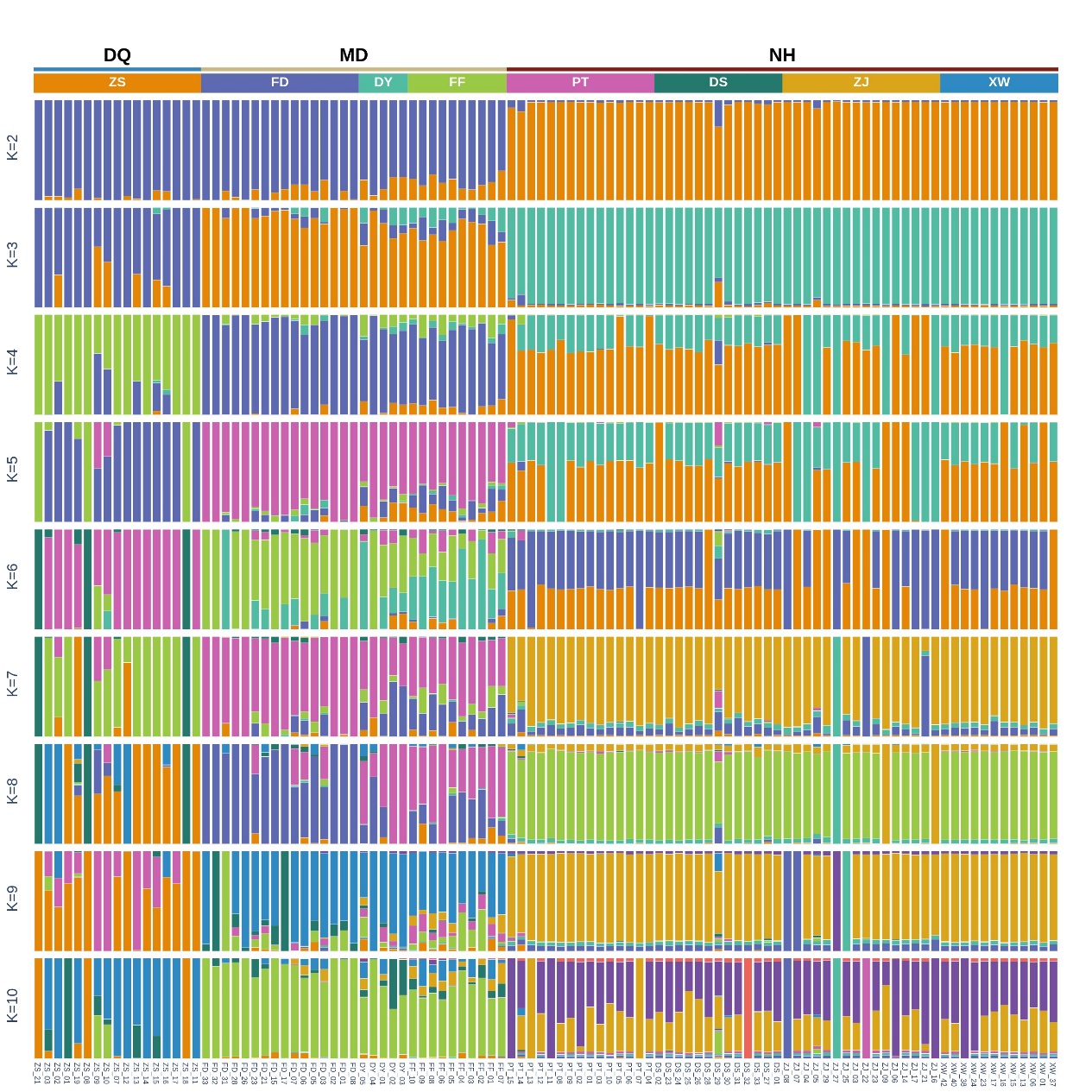


**Figure S3.** Phylogenetic tree built with maximum likelihood (ML) algorithm based on all sampled fish.


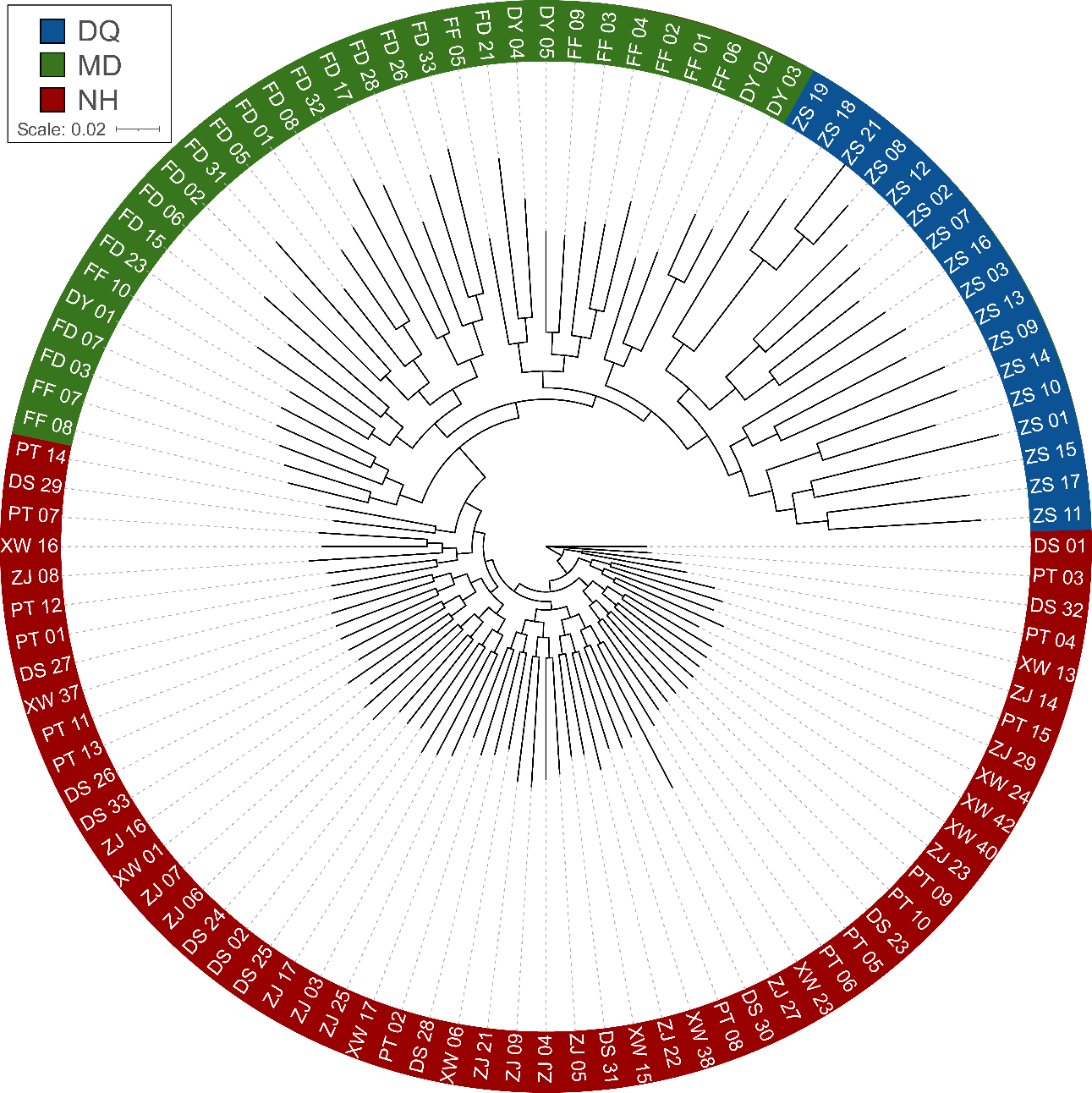


**Figure S4.** Pairwise shared haplotype length among all geographical populations of the large yellow croaker.


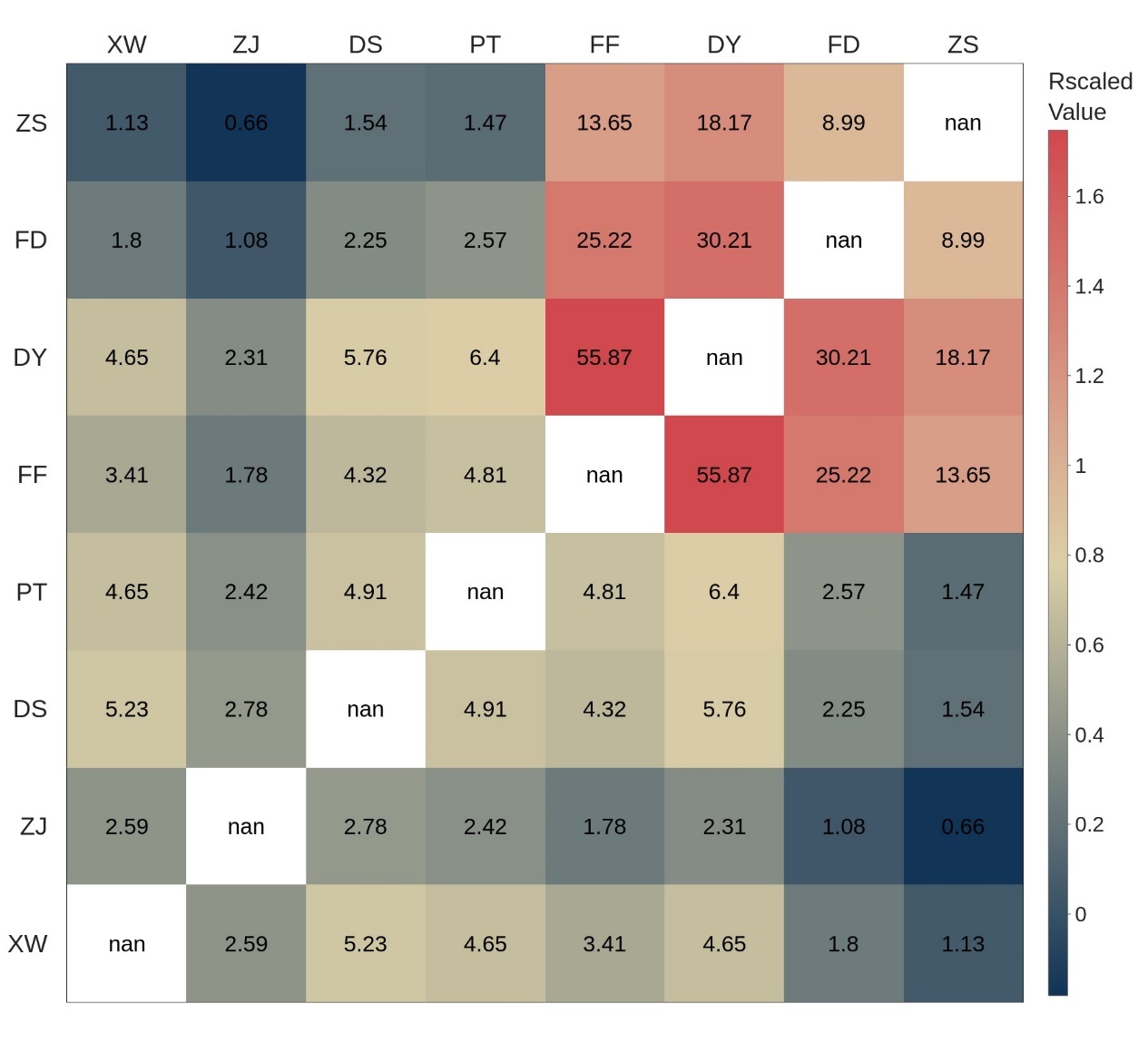


**Figure S5.** A scatter plot shows the weak negative linear correlation between the *rSHF* and *Fst* indices. Dots are colored by the three different types of natural selection they suffered in the NH stock: near-fixed (NF), recent selected (RS), and undetermined (UD).


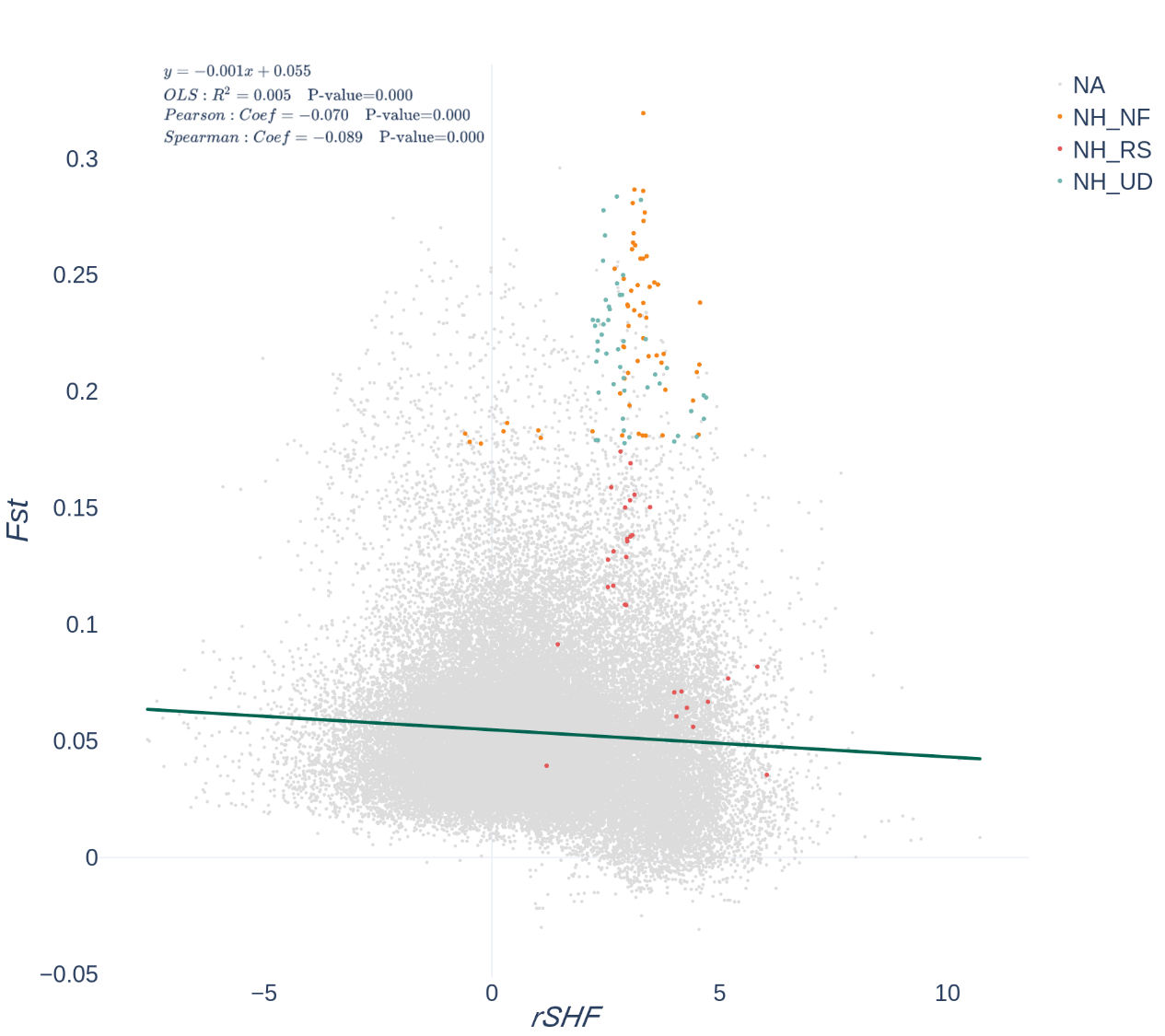


**Figure S6.** A scatter plot shows the weak negative linear correlation between the *rSHF* and *Pi* indices. Dots are colored by the three different types of natural selection they suffered in the NH stock: near-fixed (NF), recent selected (RS), and undetermined (UD).


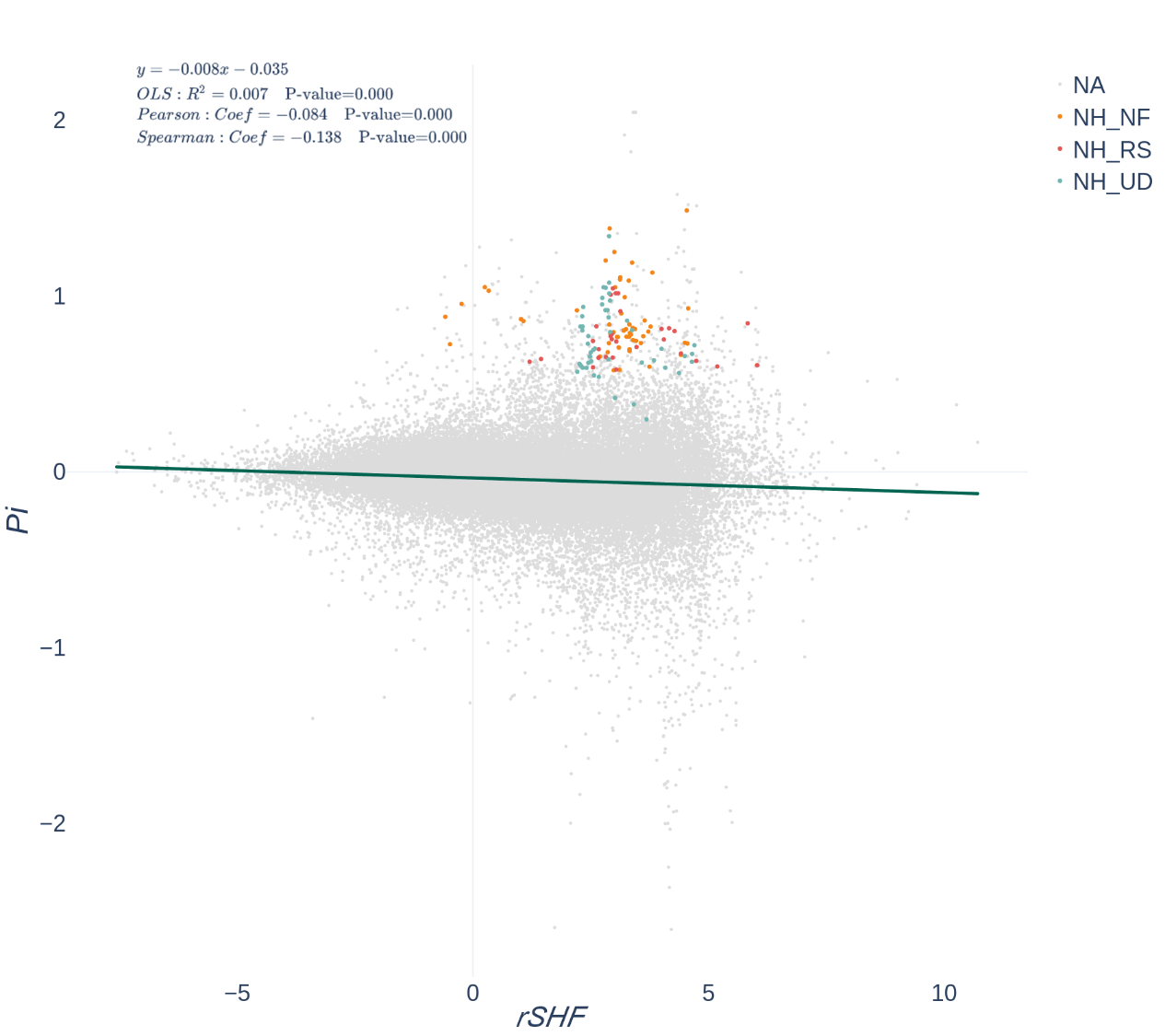


**Figure S7.** A heatmap indicates the pairwise genetic distances among large yellow croaker populations.


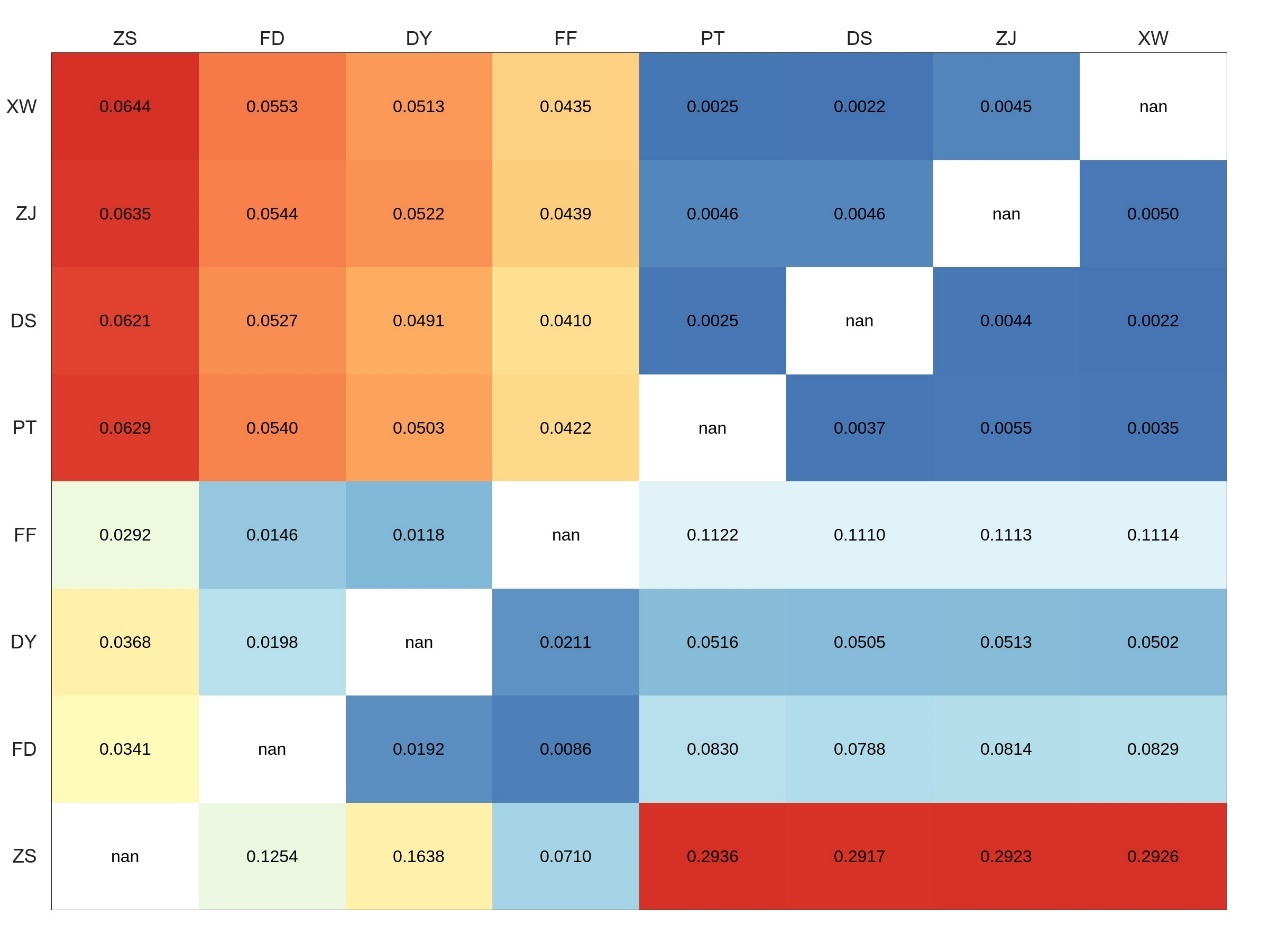


**Figure S8.** A scatter plot showing the distribution and relationship of *Pi* (x-axis) and *Rsb* (y-axis) indicesPositively selected regions (PSRs) are colored according to their *Fst* values. Histograms indicate the statistical distribution of the *Pi* and *Rsb* indices along corresponding axes.


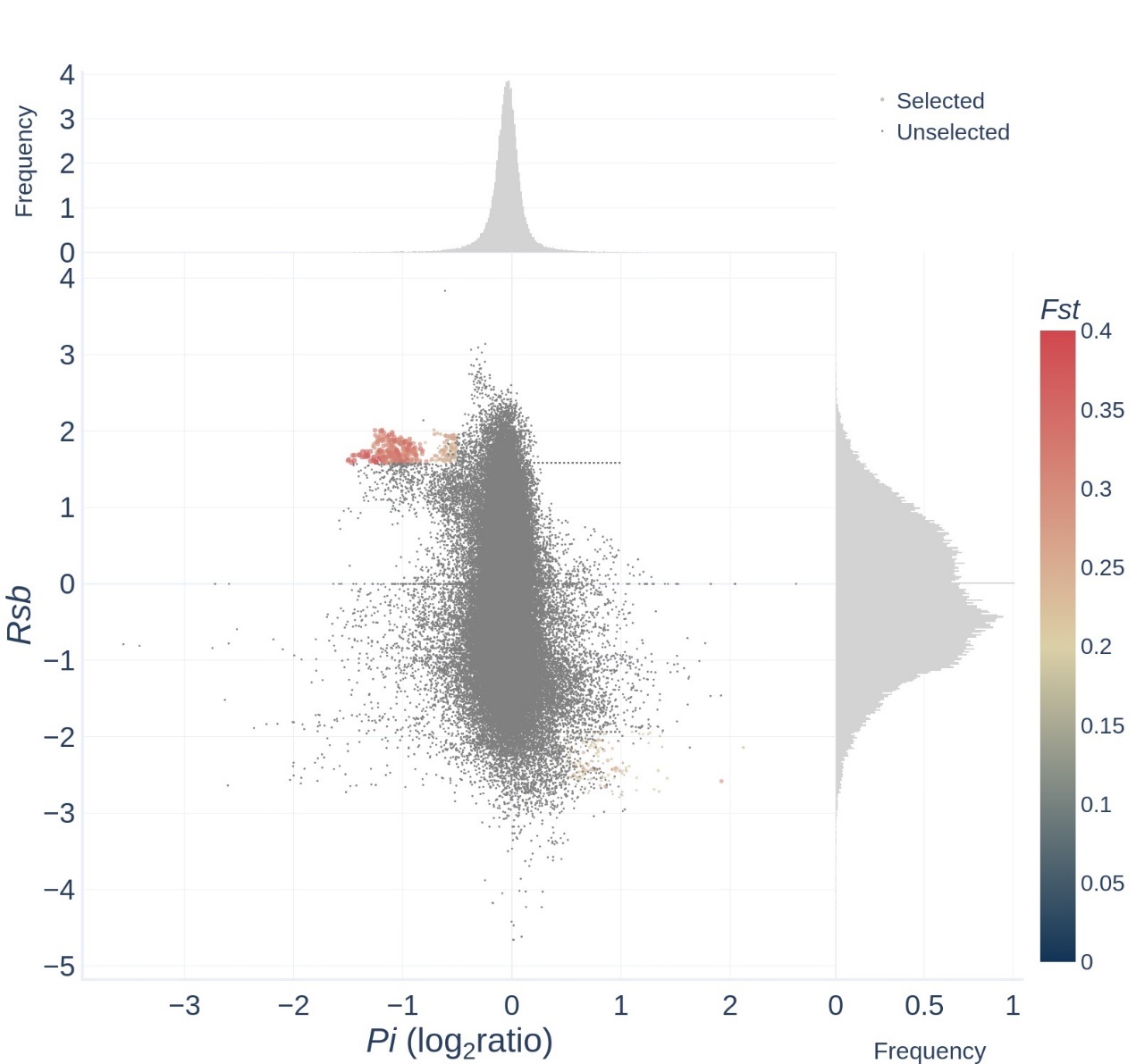


**Figure S9.** Boxplots show significant differences of five selective signatures (*Fst*, *Pi*, *Rsb*, *Tajima’s D*, and *CSS*) among different types of positively selected regions (PSRs).


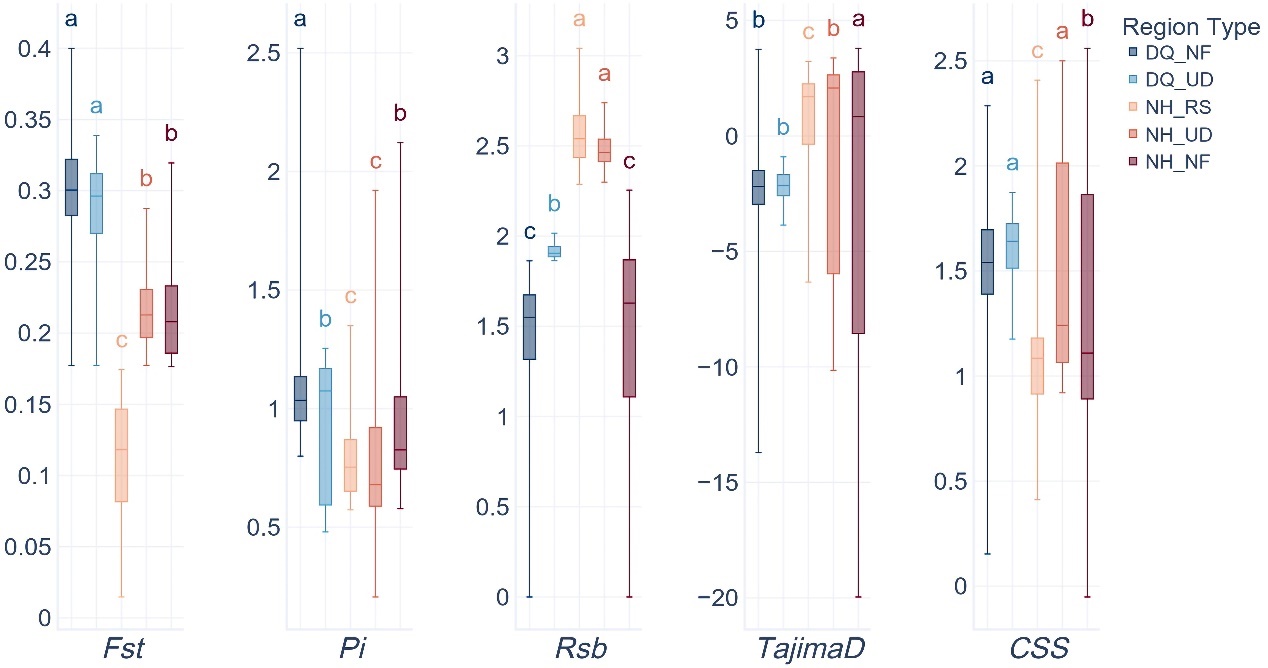


**Figure S10.** An upset plot shows the intersections among five types of positively selected genes (PSGs),


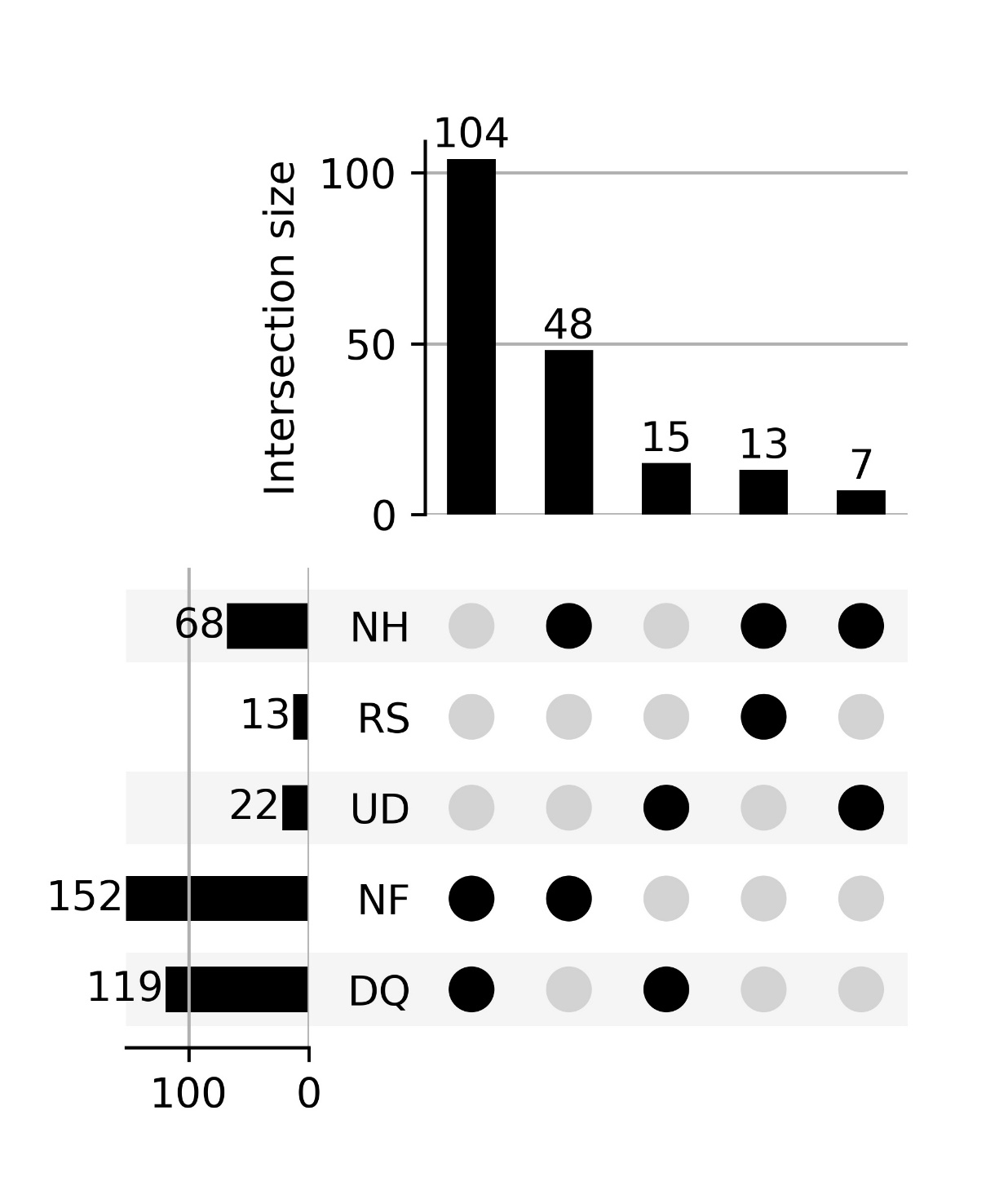

Supplement: Supplementary file 2 — A Word document includes 10 supplemental figures (DOCX 2366 KB) [file 42995_2023_165_MOESM2_ESM.docx]
